# Supplementary material for: Syntalos: a software for precise synchronization of simultaneous multi-modal data acquisition and closed-loop interventions
Source: Nat Commun. 2025 Jan 15;16:708. doi: 10.1038/s41467-025-56081-9 (PMC11736130; doi:10.1038/s41467-025-56081-9)
Supplement: Supplementary file 1 — Supplementary Information [file 41467_2025_56081_MOESM1_ESM.pdf]

Syntalos: A software for precise synchronization of simultaneous multi-modal data acquisition and closed-loop interventions

Supplementary Information

Matthias Klumpp[1], Lee Embray[1], Filippo Heimburg[1], Ana Luiza Alves Dias[2], Justus Simon[1], Alexander Groh[1,3], Andreas Draguhn[1,3], Martin Both[1,3*]

[1]Institute of Physiology and Pathophysiology, Medical Faculty, Heidelberg University, 69120 Heidelberg, Germany

[2]Brain Institute, Federal University of Rio Grande do Norte, Natal, RN 59078-900, Brazil

[3]Interdisciplinary Center for Neurosciences (IZN), Heidelberg University, 69120 Heidelberg, Germany

69120 Heidelberg, Germany

[*] Correspondence: mboth@physiologie.uni-heidelberg.de

Additional Methods

Hardware

Various hardware components were used for evaluation of the program's functions:

PC Systems:

- HP DV7-7147SG laptop with an Intel i5-3210M and 8 GB of memory
- Lenovo E495 laptop
- PC with an AMD Ryzen 5 5600G CPU and 16.0 GB of RAM
- PC with an AMD Ryzen 5 2600 CPU, 32 GB RAM, AMD Radeon RX 560
- PC with an AMD Ryzen 5 7600 CPU, 16 GB RAM, AMD Radeon RX 5500 XT

Data Acquisition Hardware:

- Intan Technologies RHD USB Interface Board with an RHD 64-Channel Recording headstage (Intan Technologies, Los Angeles, California)
- The Imaging Source Cameras: DFK 37BUX462 and DFK 37BUX290 (The Imaging Source LLC, Charlotte, United States)
- Basler ace Classic acA1920-25um camera (Basler AG, Ahrensburg, Germany)
- UVC Webcam, AnkePower Computer Webcam V-24 1080p (generic camera, Amazon online shop)
- UCLA Miniscope v4 (Los Angeles, California)
- Arduino Uno R3 (Conrad Electronic, Hirschau, Germany)
- GALDUR board, custom device, hardware design available at https://github.com/bothlab/galdur

Reference hardware:

- Cambridge Electronic Design Ltd. Micro1401-3 (Signal generator, CED, Milton, United Kingdom)
- Raspberry Pi Pico (Conrad Electronic, Hirschau, Germany)
- Kingbright DE4SGD LED (Conrad Electronic, Hirschau, Germany)
- ME/W-SG signal generator (Multichannel Systems, Germany)

Some of the data are generated by using the CED as a signal generator. However, most of it has been generated using a signal generator based on a Raspberry Pi Pico. This device can be used to test time synchronization easily and without any expensive equipment and to verify Syntalos' performance in new environments, and to calibrate it if necessary. We provide code and instructions to build this device for total costs below 10 € at https://syntalos.org/docs/timesync-verification/

Devices for behavioral experiments:

- Gates/feeder/etc as created by Lee Embray and printed with FormLabs Resin Printer (FormLabs GmbH, Berlin, Germany)

  See https://github.com/bothlab/maze-hardware for building instructions.

Data Storage & Formats

Syntalos uses a new, universal layout (EDL; 'Experiment Directory Layout', Fig. S2) for storage of different types of data together with their metadata (for details, see https://edl.readthedocs.io). EDL forms a hierarchical directory structure, where specific data are stored using suitable formats for each data type, e.g. Intan's own RHD file format for electrophysiology data, or various containers/codecs for video data. Permissible data formats are restricted to a small set of open-source formats to standardize and simplify the subsequent analysis. The EDL directory structure follows the broad logical structure of data stored in HDF5 (Hierarchical Data Format 5) files, but is a directory structure rather than a single-file format. It is similar to prior solutions like the Exdir format [1], but has been specifically designed for raw data acquired with Syntalos, which allows to use a wider variety of formats for storage of different datasets.

A single experimental recording is stored in a directory automatically named by Syntalos and is called a "collection" in EDL's terminology. Each collection can have "groups" and "datasets" as child directories. A "dataset" represents a leaf of the directory tree, containing actual data and metadata, while a "group" organizes datasets and other groups into a logical, nested structure. Any EDL organizational type is referred to as a "unit" (Fig. S2A). Syntalos will automatically sort recorded datasets into groups and name both items according to its default rules, using the names of the data-generating modules as base. However, experimenters can change the naming as they like. Data may be unprocessed raw data or may be generated by an analysis module, e.g. positional information from a behavioral experiment (see example in Fig. S2B). Metadata are automatically generated by Syntalos, depending on the recording module, and stored in a well-defined structure as specified for the EDL format. An EDL "collection" (equivalent to a single experiment run) is assigned a unique ID that is also written into individual recorded data files. This allows attributing data files to a specific experiment, even if they were taken out of the EDL directory structure. For offline analysis, we created a Python module named 'edlio' (https://pypi.org/project/edlio/) that supports reading Syntalos-recorded data from an EDL collection and can create new EDL units for analysis results.

For timing information, Syntalos uses *tsync* files. This Syntalos-specific file format stores two time series, one of the master clock and one of a secondary clock (the clock of the synchronized device) in a binary format together with metadata. These include the EDL UUID (universal unique identifier), time unit (e.g., milliseconds), and integer data types for the individual time values. A *tsync* file can exist in two modes: 'Continuous' and 'SyncPoints'. In 'Continuous' mode, each time point of the first clock is matched exactly with one time point of the second clock with no gaps (e.g. matching a timestamp of the Master clock with a video frame number). In 'SyncPoints' mode, only some timestamps are mapped to the secondary clock, while the

missing timestamps need to be approximated, e.g. by linear interpolation, in post-processing (e.g. master clock time of a sample to device clock time of a sample). This mode is most suitable for very high frequency data sources, while the continuous mode is best for data sources with low to medium sampling rate.

The individual timestamp data is chunked into blocks of a sampling-rate dependent size. Each block is hashed using the XXH3 fast non-cryptographic hash algorithm (Collet), with the hash stored after each block with a terminator sequence. This simplifies data recovery in case the binary file gets corrupted (e.g. in transmission or by broken hardware), as the exact 'bad' block can be pinpointed and ignored. In this case, only one block is lost instead of having an entirely broken file. The risk of silent data corruption is also reduced. Therefore, *tsync* binary files can be as robust as CSV files while being much faster to write and read. The 'edlio' Python module contains a built-in reader for *tsync* files that handles them transparently, but Syntalos also provides a small tool to convert them into a textual CSV-like representation if needed.

Comparison of the EDL data storage layout to other data formats

HDF5: The Hierarchical Data Format (HDF) is a file format to store large amounts of data in a single, structured file. Currently, HDF5 is the most widely used container format for a variety of other file formats, and is especially useful to store array data. While HDF5 is an excellent format to store structured data, and has the advantage of being just one file instead of a directory structure like EDL, it also has some disadvantages which may make EDL preferable in some applications:

- Writing in parallel to multiple datasets in a HDF5 file is very slow due to thread synchronization, limiting HDF5's usability for massively parallel data acquisition.
- We observed that in case of an error while writing (e.g. due to a crash of the writing process), the entire HDF5 file may be corrupted, instead of just one file. This may happen in live recordings for a variety of reasons, in which case a lot of data is lost. EDL, by comparison, is more robust towards errors and easier to recover.
- HDF5 has a limited set of ways to compress its array data. EDL, by using file-formats designed for the recorded modalities, can compress the stored data more efficiently (e.g. by using the FFV1 video codec for video data).

Advantages of HDF5 are the single file structure, its wide industry support and the availability of a single library to load the entirety of an HDF5 file (while EDL relies on other tools to read the specific data, e.g. FFmpeg or Neo).

Exdir: The Experimental Directory Structure (Exdir) is an open file format specification for experimental pipelines. Like EDL, it is modeled using the same abstractions that HDF5 uses as well.

Exdir and EDL share multiple similarities, but also have several key differences:

- EDL uses TOML for metadata and Exdir uses YAML. While YAML is great to write for humans and sometimes less verbose than TOML, it has quite a lot of ambiguities

and pitfalls (see https://noyaml.com/) for humans and programs to run into, which sometimes makes it more difficult to be used for structured data storage.

- Exdir adheres fairly strictly to the HDF5 abstract data model and the data types that HDF5 supports. While arrays are well-supported as Numpy arrays, there are no standards for "raw" datasets (containing videos or images). For use in a DAQ system like Syntalos, the "raw" dataset type would have to be massively extended.
- Each Exdir dataset can only contain one format, while EDL supports "auxiliary data" that describes the contained data further and is not pure textual TOML metadata. This auxiliary data may, for example, be timestamps for a video file. In Exdir, this data would have needed to be split into two datasets, which we did not want to do. EDL allows strongly linked data to be kept together in one directory.
- Exdir has no unique ID for the dataset, and also does not mandate the ID to be added as metadata to files contained in its structure. This is a feature that we wanted for EDL, and that was difficult to retrofit into Exdir.

NWB: The "Neurodata Without Borders" (NWB) format is a HDF5-based file format and structure to store neuroscientific data and make it easy to be interchanged between many groups in the neuroscientific community. Being based on HDF5, NWB is not very suitable for massively parallel direct-write data acquisition though, which is why Syntalos and most other DAQ systems cannot use it directly.

However, EDL can be converted into NWB-HDF5 files after the data acquisition has been completed, which is a good approach to get the best of both worlds.

System Requirements

Syntalos was tested extensively on x86-64 systems and requires a Linux-based operating system with a kernel higher than or equal to version 4.20. We recommend at least 8 GB of memory and a CPU that implements the AVX2 x86 instruction set extension. In general, any CPU supporting the x86-64-v3 feature set (released in 2015) should be fine for Syntalos. The system with the lowest specifications on which we tested Syntalos was a HP DV7-7147SG laptop (released circa 2012) with an Intel i5-3210M (2 cores, 4 threads) CPU and 8 GB of memory. The system with the highest specifications contained an AMD Ryzen 5 7600X (6 cores, 12 threads) CPU and 16 GB of memory. The tested operating systems were Ubuntu 22.04, Ubuntu 24.04, Debian 12 and Debian 13 (development version). Syntalos can use a suitable GPU to offload video encoding tasks, and some of Syntalos' modules may even use OpenCL for acceleration. In general though, a powerful GPU it is not required. In our experiments, we used an AMD Radeon RX 560 and an AMD Radeon RX 5500 XT.

The precise system requirements for Syntalos depend strongly on the individual experimental setup, e.g. how many cameras are in use and how much data processing has to happen online and in parallel. Syntalos is particularly sensitive to CPU performance, disk I/O performance and RAM clock speed (ordered according to relevance). Another important factor for optimal data acquisition is the available interfaces. Many USB devices only have optimal bandwidth and latency if connected to an individual USB controller. Syntalos provides a diagnostic tool to

help users find optimal USB ports for devices using the USB protocol. In summary, it is important to have a good CPU, more than 8 GB of memory, a fast disk, and multiple USB controllers. Syntalos is able to display extensive information about the system hardware via its diagnostic panel.

Module development and expandability

To extend Syntalos with user-defined experiment-specific algorithms such as the logic of cue presentations and gate openings in a behavioral task, some programming knowledge is required. Most of such tasks can be accomplished using Python code. In certain cases like expanding hardware support or implementing performance-critical modules C/C++ knowledge may be advantageous. Several existing DAQ solutions such as Bonsai RX [2] make use of built-in graphical programming interfaces. Such GUIs make it possible to customize data acquisition without using a text-based programming language, especially for relatively simple designs. However, basic Python programming abilities are widespread in the scientific community [3]. We therefore opted for Python programming which, in our experience, is superior for more complex experimental designs. Furthermore, Syntalos imposes no limitation on the scripting languages, provided a C++-based integration module is present for it. In the same way, it is possible to implement a Syntalos module that leverages a graphical programming language such as the one used by Bonsai RX.

Syntalos runs on Linux. This may hinder its adoption by users of other operating systems. It allows, however, inspection of the whole data analysis pipeline in source code, precise analysis of program performance, and easy integration with existing Linux-based Python code. We are convinced that these advantages outweigh the potential drawback of being Linux-specific. It is also well possible to port Syntalos to other operating systems which does, of course, require some computation skills and careful performance validation.

Comparison to other software solutions

When comparing with proprietary solutions like ANY-Maze, EthoVision and LabVIEW, Syntalos can not offer the dedicated hardware that these vendors offer for sale that is tuned to their software, and also does not offer paid support. However, being open-source allows engineers and scientists to extend the software on their own, or contract anyone to do so instead of being dependent on a single vendor. Syntalos' support for open-source hardware and hardware from select vendors who offer open APIs is excellent, but so far Bonsai has a few more modules for hardware integration available and a larger community behind it. All of the tools have some method for custom programming, with Bonsai, LabVIEW, EthoVision and ANY-Maze offering a graphical programming language, while Syntalos currently only has Python, MicroPython and C++ scripting abilities. Graphical programming could be added to Syntalos as a module in future. Bonsai and Syntalos both offer good support for Python, with Syntalos [due to using CPython (python.org) instead of IronPython (ironpython.net)] making it a lot easier to run preexisting Python code as a module, while code for Bonsai may need adjustments. Of all of the tools, Syntalos currently is the only one with consistent raw data management built into its core, while the other tools allow a variety of formats and data export methods (see Table S4). Syntalos is also the only tool providing fully automated, algorithmic

time synchronization. We compare Bonsai and its synchronization performance to Syntalos in detail in the Results and Fig. S5.

With the exception of PyControl (https://pycontrol.readthedocs.io/en/latest/), AutoPiLot (https://auto-pi-lot.com) and LabVIEW, none of the existing solutions run on Linux or have been specifically developed to make use of its capabilities, though an effort to port Bonsai to Linux exists.

Syntalos is well-equipped to integrate into the existing landscape of neuroscience data handling tools. It provides a directory structure to save data together with metadata in an entirely machine-readable format which enables easy translation of all raw data into commonly used standards like NWB [4] or G-node [5]. In addition, Syntalos stores all data in open-source file formats that can be read with standard tools available to neuroscientists, such as Ephyviewer for electrophysiology data [6], ImageJ/Fiji for images [7] or simple media players for video files. The use of open-source formats avoids licensing costs, and the resulting data are readable by every lab in the foreseeable future. The edlio Python module (https://edl.readthedocs.io/latest/) facilitates integration of Syntalos-acquired data into existing Python-based analysis pipelines. In summary, data acquired and stored with Syntalos are usable in a highly versatile way and can be integrated into a broad variety of existing tools.

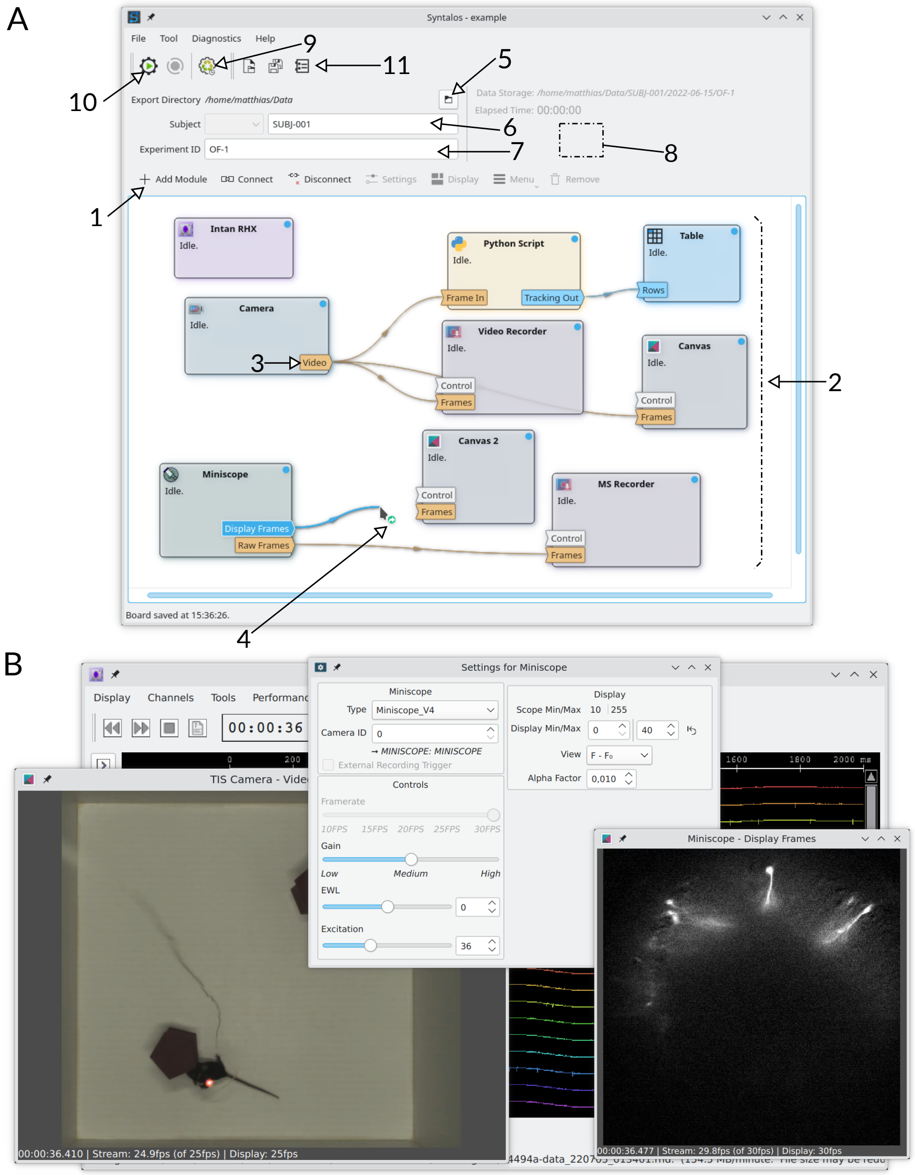

Figure S1: Syntalos UI. A: A screenshot of the Syntalos main control window. The "Add Module" button (1) allows the addition of a new module which is represented in the connection view (2) as a rectangular box. A module has ports (3) which allow piping data out of a module or feeding it data. Input ports are on the left side, while output ports are on the right side of the module rectangle. Modules can be connected by the user via simply dragging a line from an output port to an input port (4). To assess the position of an animal in an experiment, for example, the output port of the video camera module can be linked to the video input port of a position detection module. Once a line exists, data from one module will automatically flow to the other module. The direction is indicated by a small arrow on the connecting line itself. To export data, a directory to store the recording needs to be selected (5), the tested subject can be given a name by selecting one that was previously defined from a dropdown list or entering one manually (6). The experiment can be given a user-defined name as well (7). During a run, Syntalos will display information about the current run, like the save path, elapsed time and information about any issues on the right-hand panel (8). Once an experiment is configured, a test run can be started (9) where no data will be permanently saved, or a real recording can be launched (10). In addition to basic configuration, the experimenter can also set additional project-specific settings via the "Project" button (11). B: Some open control and display windows for the depicted modules. From left to right in the foreground: Canvas displaying a live video of the animal, Controller for Miniscope recording parameters, Canvas displaying background-subtracted live Miniscope recording, Intan RHX electrophysiology display & recording controller in the background.

Icons/logos reproduced with permission (see Acknowledgements).

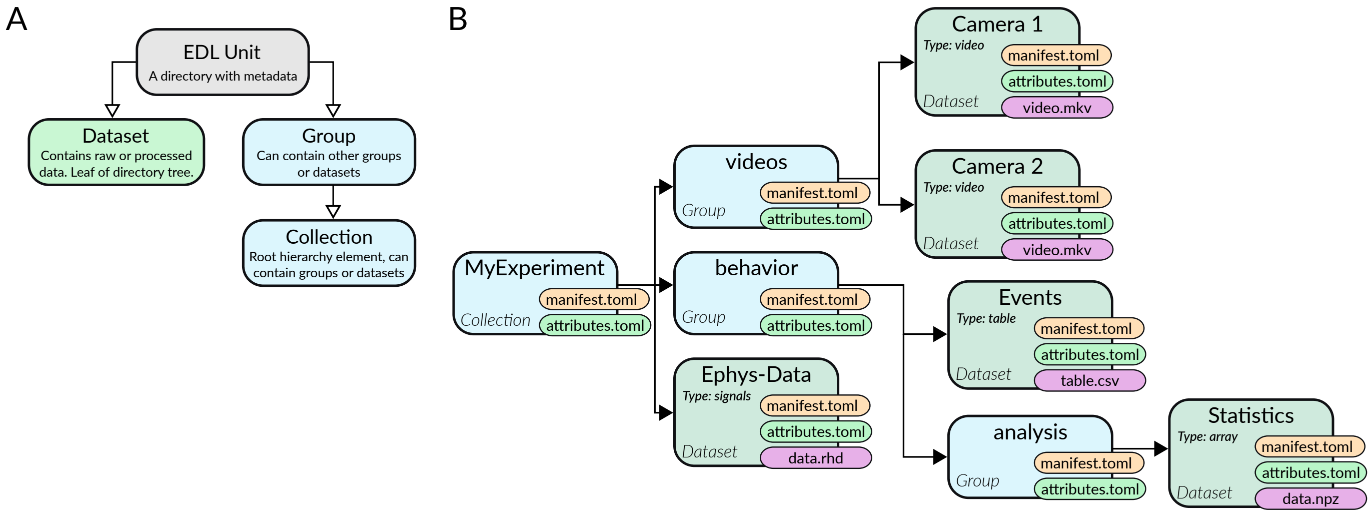

Figure S2: EDL data storage scheme. A: Organizational structure of EDL (Experiment Directory Layout) elements. A basic EDL unit is a directory with metadata in the TOML format (manifest.toml, optional: attributes.toml). An EDL group contains other groups or datasets. An EDL collection is the root of a directory tree and represents one experiment run recorded by Syntalos – it sets the unique ID of the experiment and contains metadata like the experimenter's name and experiment duration. A dataset is an EDL unit containing the actual recorded data with its metadata. It is a directory tree leaf and may not contain more nested datasets or other EDL units. B: Concrete example of a recorded experiment. Each node represents a directory with the given name (e.g. "videos"). Each directory contains metadata in the mandatory "manifest.toml" files and optional "attributes.toml" files for user- or module-defined metadata. A dataset name (= directory name) is derived from the Syntalos module name which can be set arbitrarily by the user in Syntalos' GUI. No two modules with the same name are allowed to be created. The amount of data types datasets may contain is limited, so data parsing is simplified. For some datatypes, specific rules are applied (e.g. number formatting rules in CSV files, and semicolons being used for value separation) to increase consistency and ease data parsing. In this example, data analysis has been performed for the dataset in an "analysis" EDL group and integrated into the EDL structure. EDL exists for Syntalos to easily and quickly store data in parallel with all metadata and auxiliary data (timesync info, timestamps, …) included. It can be used by experimenters to store their analysis data as well, but this is not required. Experimenters may convert the EDL structure into anything they want and generate analysis data in any format they want (e.g. NWB for neuroscientific analysis data).

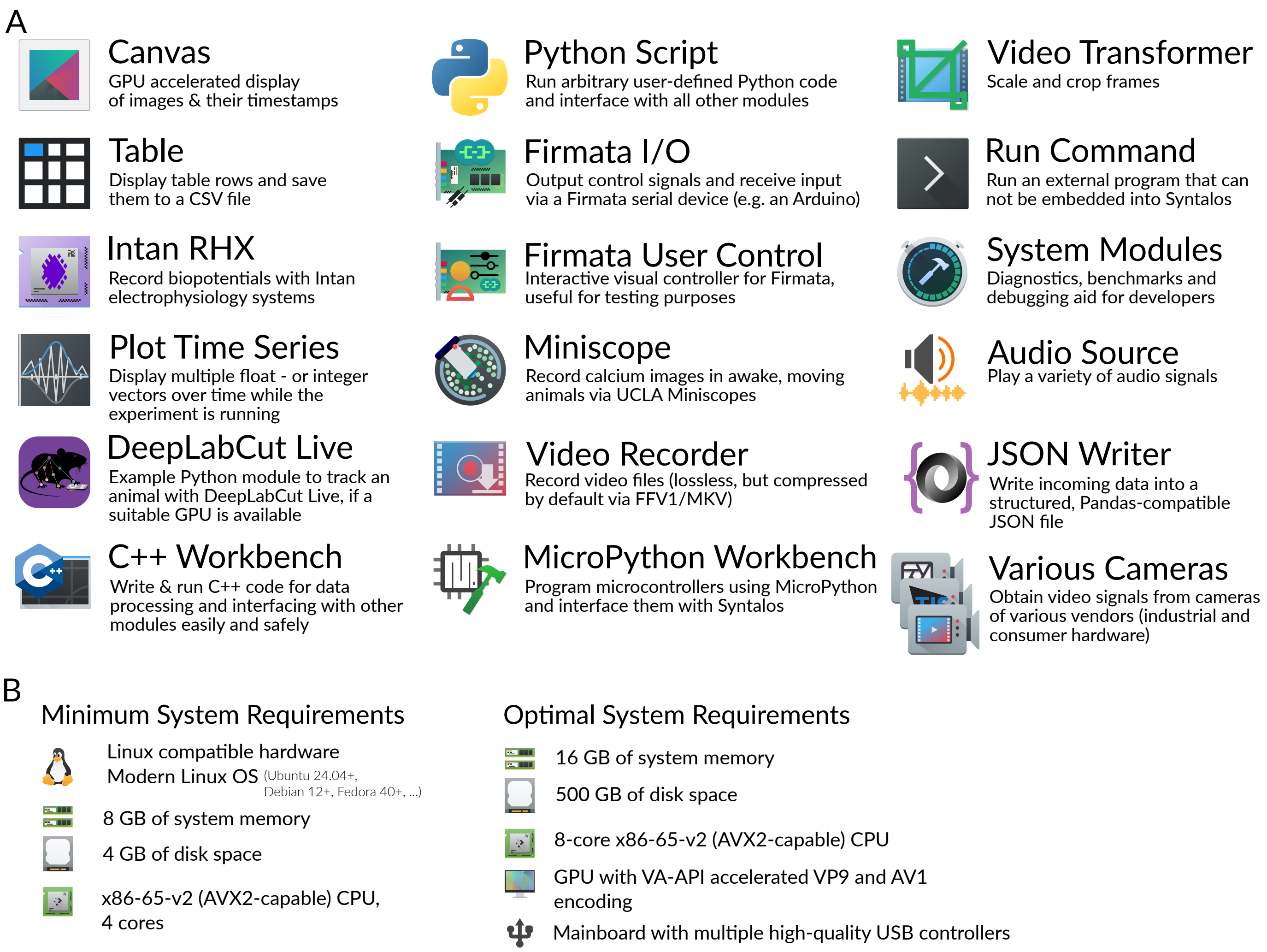

Figure S3: Syntalos Module Overview and Hardware Requirements. A: A list of the most important readily available Syntalos modules and their respective functions. New modules can be implemented by the user in either C++ or Python. B: System requirements as well as hardware recommendations for new data acquisition setups using Syntalos.

Icons/logos reproduced with permission (see Acknowledgments).

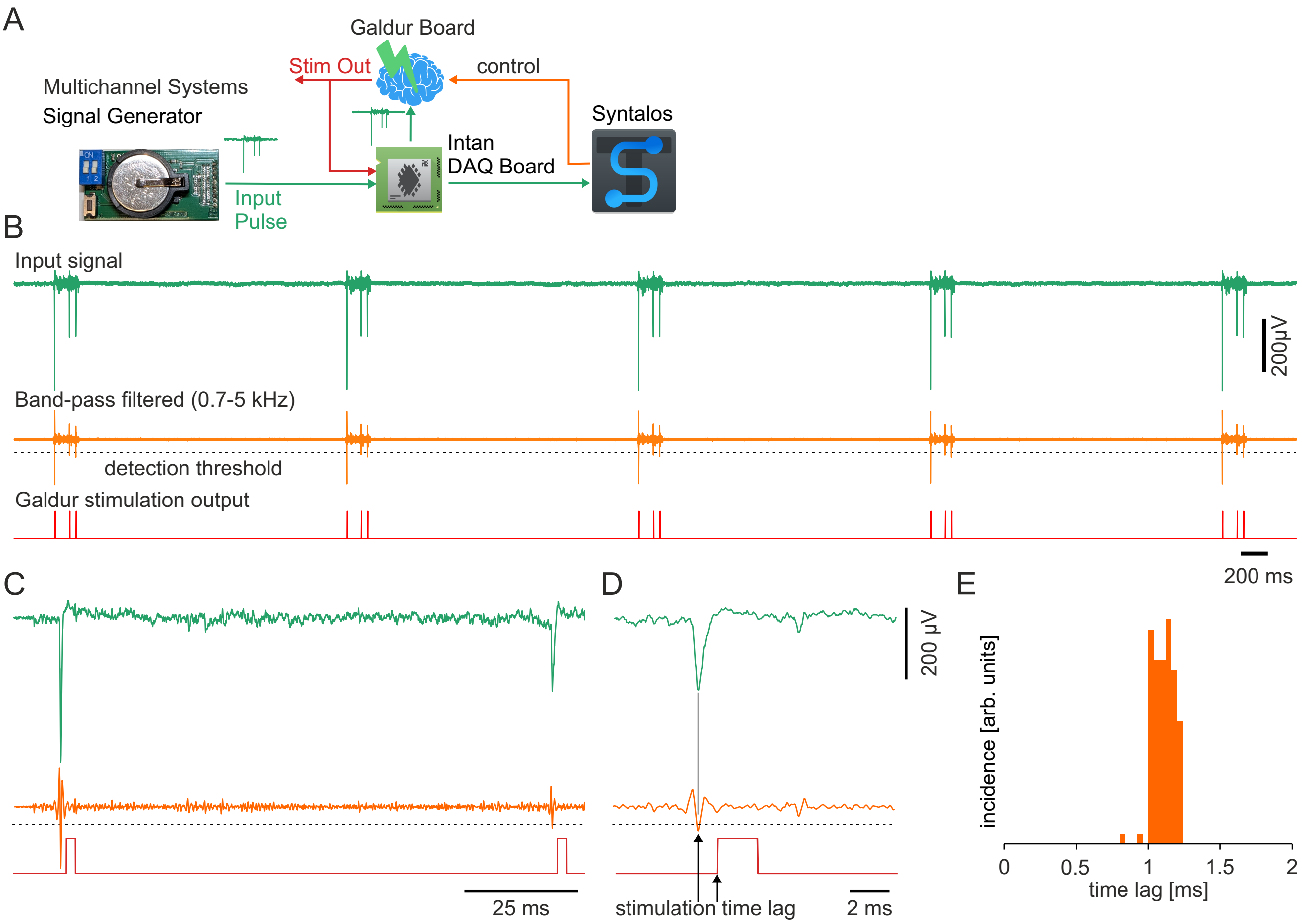

Figure S4: Fast closed-loop experiments with the dedicated Galdur board Hardware. A: Schematics of the evaluation of closed-loop reaction time. A signal generator (ME/W-SG, Multichannel Systems) simulating extracellular spikes of hippocampal pyramidal neurons is connected to the Intan DAQ board controlled by Syntalos. The recorded signal is routed to an analog output and fed into the Galdur board which itself is connected to a Raspberry Pi 4, where it is processed and a stimulus is generated under user-defined conditions. B: For this experiment, stimulation is performed if an extracellular spike is detected. The Raspberry Pi runs a C++ routine that digitally band-passes the signal (0.7-5 kHz) to filter out low frequencies as well as high frequency noise. Then, a threshold is applied to the signal and a stimulation TTL pulse is generated if the threshold is crossed. C and D: Close-up of B. E: Distribution of the latency between a spike trough and the stimulus output. Note that this latency is mainly dependent on the signal length that has to be evaluated by the digital filter. For lower frequencies or frequency contents (e.g. ripple oscillations, ~200 Hz, i.e. 5 ms per cycle), the latency will be longer. Icons/logos reproduced with permission (see Acknowledgements).

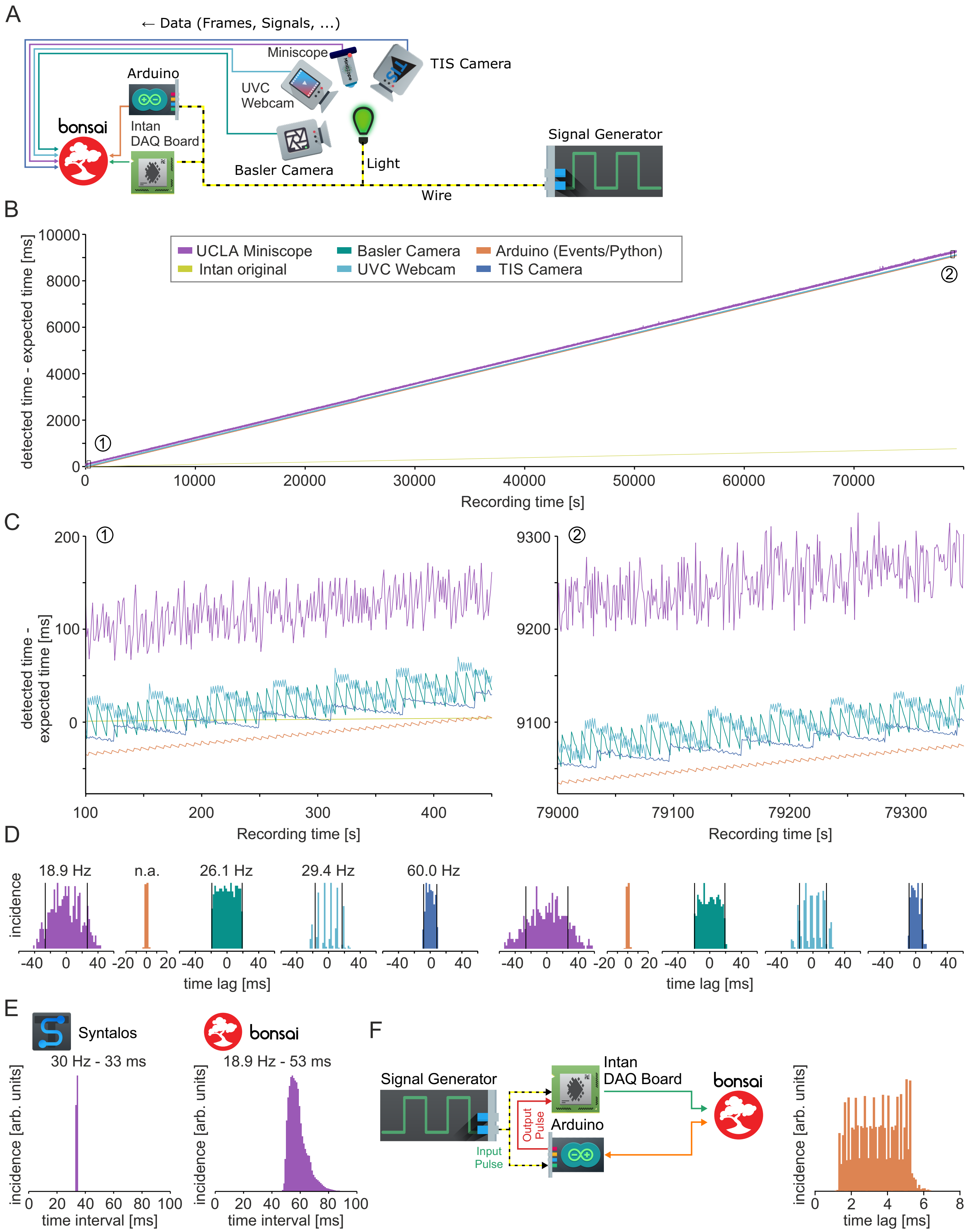

Figure S5: Synchronization performance of Bonsai. A: Experimental setup similar to Figure 3. Due to missing drivers, not all devices shown in Figure 3 could be compared. In this configuration, we used a standard USB UVC (Universal Video Capture) webcam (intended sampling rate 30 Hz), two different scientific cameras (Basler, intended sampling rate 26 Hz; The Imaging Source, intended sampling rate 60 Hz), a UCLA Miniscope (intended sampling rate 30 Hz), an Intan RHD2000 electrophysiology USB

interface board (sampling rate 20 kHz), and an Arduino Firmata I/O serial interface connected via USB. B: Time deviation of the recorded timestamps from the expected time points. Note that the Intan device is much faster than the other clocks which adds up to an error of more than 9000 ms after 22 hours of recording. C: Close-up of an early time point (indicated by 1 in panel D) and a late time point (indicated by 2 in panel D) of the recording. D: Distributions of the timestamps of the various devices relative to the low-pass filtered timestamps of the TIS camera (fastest device). Left panels are from the time window depicted in the left panel of C, and right panels from the time window depicted in the right panel of C. The timestamps of the devices stay mostly within their expected range i.e. within their frame rate limits, with the exception of the UCLA Miniscope. Also, expected frame rates are within the range of their intended sampling rates, again with the exception of the UCLA Miniscope. E: comparison of the inter-frame intervals of the UCLA Miniscope recorded with Syntalos (left panel) and with Bonsai (right panel). F: Distribution of the latency between a Bonsai-triggered output command and the TTL pulse generated by the Arduino board (similar to Figure 5D). Icons/logos reproduced with permission (see Acknowledgements).

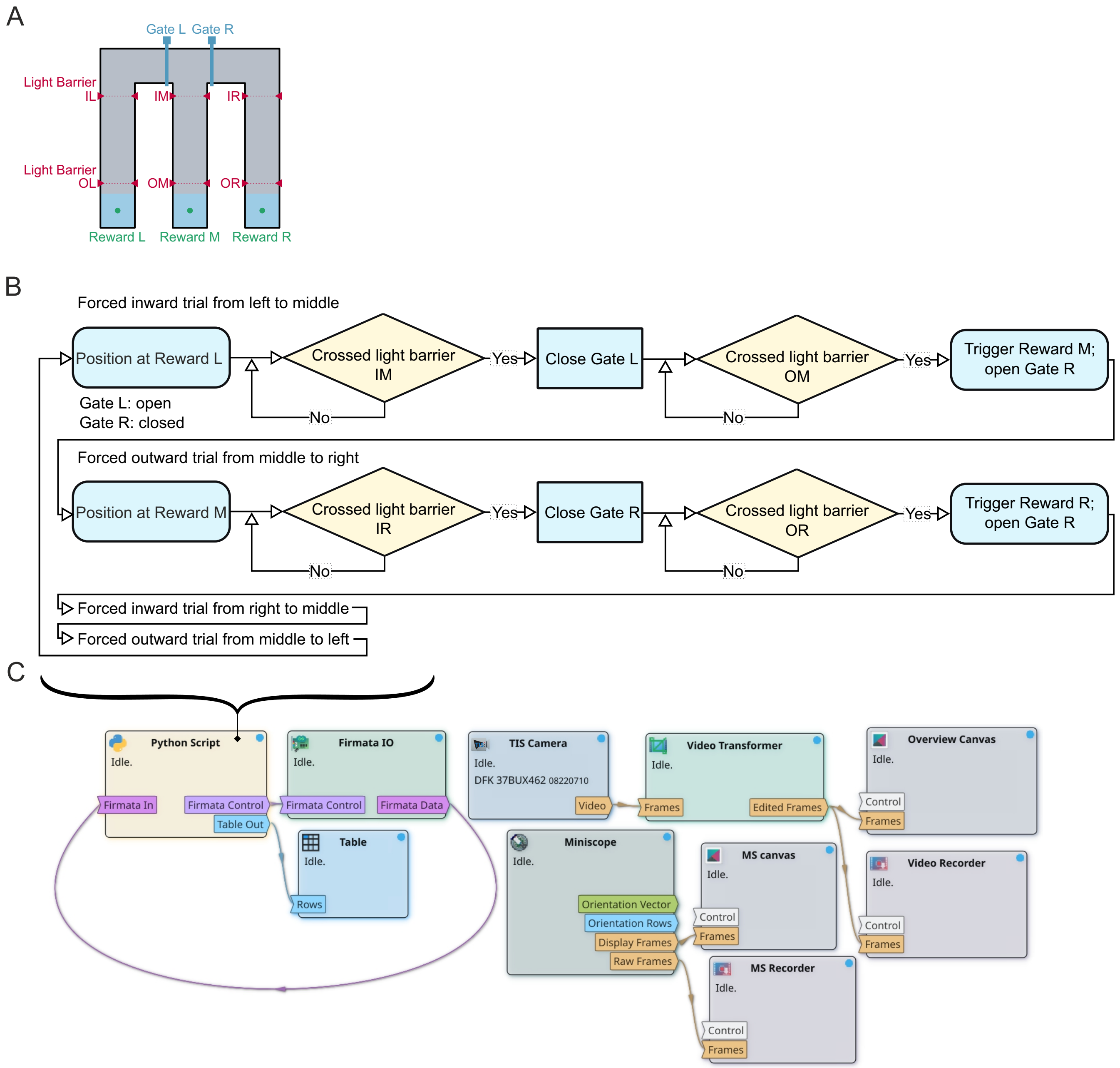

Figure S6: M-maze logic and Syntalos module settings. A: Schematics of the detectors (light barriers) and actuators (automated gates and automated food dispensers) of the M-maze. B: Logic of the behavioral experiment for forced trials (during the familiarization phase), which is implemented in the Python scripting language and controls an Arduino board running Firmata IO. C: Syntalos modules required for this experiment and their connections. Icons/logos reproduced with permission (see Acknowledgements).

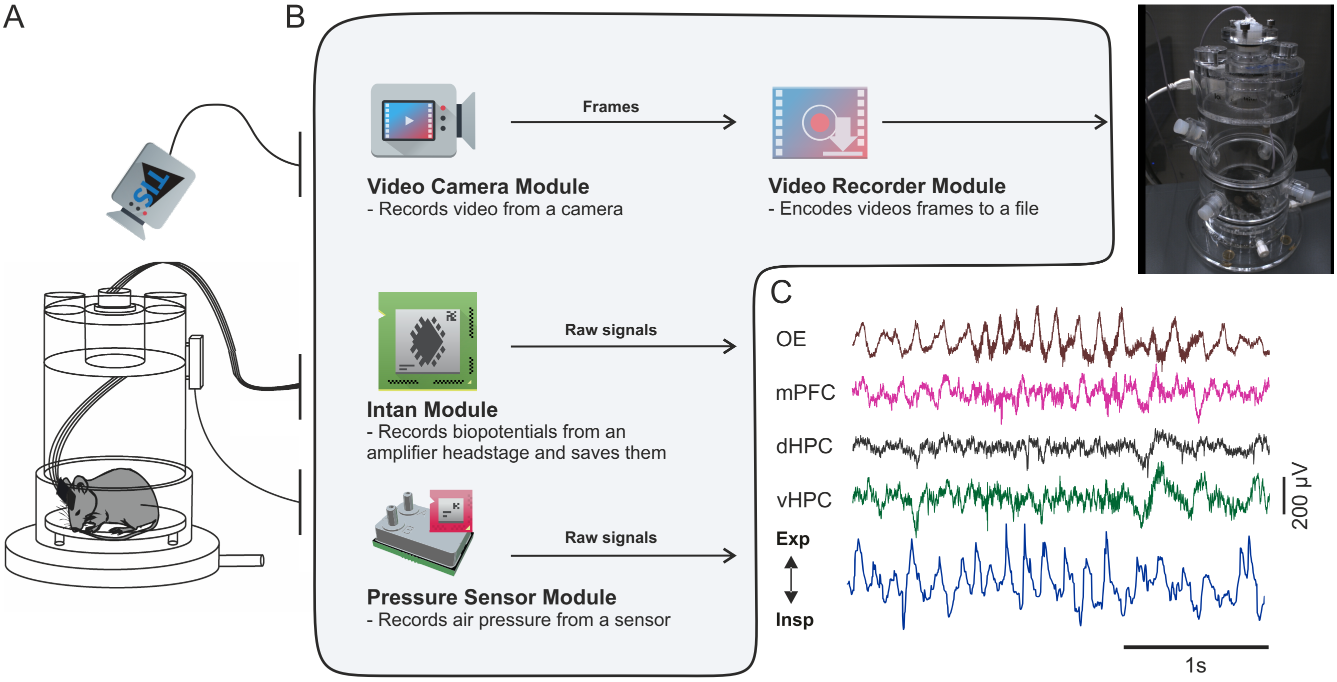

Figure S7: Application example. A: Schematics of a behavioral experiment where local field potentials from various brain structures are simultaneously recorded with the respiration state assessed by a plethysmograph. B: Modules used for this experiment. C: Respiration state (upper panel) and local field potentials (four lower panels) recorded in the olfactory epithelium (OE), the medial prefrontal cortex (mPFC), and the dorsal (dHPC) and ventral (vHPC) hippocampus. Icons/logos reproduced with permission (see Acknowledgements). Panel A reproduced from [9] with permission.

Supplemental Tables

Table S1: Data types that can be transferred between modules.

Data Type	Purpose
ControlCommand	A command (Start, Stop, Pause, Step, …) that one module can send to another to change its state.
TableRow	A row of a (CSV) table as text
FirmataControl	A Firmata API control command (such as registering a new digital pin, or writing to one)
FirmataData	Data returned by read operations on a device using the Firmata protocol.
IntSignalBlock	A block of data consisting of a matrix of integers.
FloatSignalBlock	A block of data consisting of a matrix of doubles.
Frame	A single frame recorded by an imaging device in form of an OpenCV matrix.

Table S2: Third-party software used for Syntalos core.

Name	Version	Comment & Purpose	Reference
GCC	10.2	C/C++ compiler	gcc.gnu.org
Qt (Core, GUI, Test, OpenGL, SVG, D-Bus, Concurrent)	5.15	GUI toolkit and basic functionality	www.qt.io
xxhash	0.8.1	XXH3 hash for error-detecting codes	cyan4973.github.io/xxHash
Eigen3	3.3.9	Linear algebra & matrix operations	eigen.tuxfamily.org
toml++	3.2.0	TOML format read & write support	marzer.github.io/tomlplusplus/
OpenCV	4.5.1	Data types & basic operations for image processing	opencv.org
readerwriterqueue	~	Embedded, modified. A fast lock-free queue	moodycamel.com/blog/2013/a-

		implementation for multithreading	fast-lock-free-queue-for-c++.htm
Libusb	1.0.24	Direct USB support	libusb.info
Iceoryx	2.0.6	Low-latency IPC	iceoryx.io
Dear ImGui and ImPlot	1.90	Immediate-mode plotting	github.com/ocornut/imgui & github.com/epezent/implot
systemd	252.6	Hardware support, system state read/write (e.g. for changing sleep states)	freedesktop.org/wiki/Software/systemd/
FFmpeg	5.1.3	Video encoding	ffmpeg.org
GLib2	2.66.8	C utility functions, Event loop	wiki.gnome.org/Projects/GLib
KArchive	5.78	TAR/ZIP read/write support	invent.kde.org/frameworks/karchive
KDBusAddons	5.78	D-Bus convenience functions	invent.kde.org/frameworks/kdbusaddons
KConfigWidgets	5.78	UI preferences, color scheme support	invent.kde.org/frameworks/kconfigwidgets
GStreamer	1.18	Media processing pipelines, audio generation	gstreamer.freedesktop.org
Python	3.11	Simple high-level programming language	www.python.org
pybind11	2.6.2	Interoperability between Python and C++	pybind11.readthedocs.io
Breeze Icons	~	Icon / color theme and design language used by Syntalos	invent.kde.org/frameworks/breeze-icons
Flatpak	1.14.4	Simple deployment of Syntalos to any Linux OS	flatpak.org

Table S3: Third-party software used for additional hardware support.

Name	Version	Purpose	Reference
PoMiDAQ	0.4.5	For UCLA Miniscope support, uses libminiscope	github.com/bothlab/pomidaq
Spinnaker SDK	~	Proprietary component for FLIR camera support	www.flir.de/products/spinnaker-sdk/
tiscamera	1.1.0	Open source support for The Imaging Source industrial cameras	github.com/TheImagingSource/tiscamera
Intan RHX	~	Embedded Intan RHX software for control of Intan devices	intantech.com
OpalKelly Front Panel	~	Proprietary FPGA interface library used by Intan RHX module	opalkelly.com/products/frontpanel
QFirmata	~	Embedded, modified code originally from the QFirmata project. Used for Firmata with serial interfaces	github.com/callaa/qfirmata
Firmata for Arduino	~	Arduino firmware implementing the Firmata protocol, needed on an Arduino for use with the Firmata I/O module	github.com/firmata/arduino

Table S4: Comparison of Syntalos with existing DAQ tools.

Tool	**Syntalos**
Open Source	Yes, free
Synchronization Method	Statistical algorithms for alignment to computer-clock, TTL pulses, hardware
Hardware Support	Variety of industrial cameras, Arduino, Intan electrophysiology hardware, extension possible via custom modules
Programming Model	Programmable in Python (via CPython), more extension possible in C++. Any programming language can be added as module, modules can be created in any language that supports the C FFI (Foreign function interface).
Data Standardization	Common open source data formats in an EDL directory layout.
Tool	**ANY-Maze**
Open Source	No, paid license
Synchronization Method	Manual / TTL Pulses
Hardware Support	Various cameras, custom ANY-Maze specific hardware and adapters available on their website for purchase
Programming Model	Graphical block-based programming, tightly integrated with the system.
Data Standardization	XML and CSV export, also export to GraphPad, SPSS, dBase and SYLK support. No default layout.
Comment	Has extensive animal tracking, for which Syntalos needs DeepLabCut and which is less well integrated in Syntalos. Also does a lot of data analysis and statistics which is out of scope for Syntalos.
Tool	**Noldus EthoVision XT**
Open Source	No, paid license

Synchronization Method	Unknown, likely manual/TTL-pulse based
Hardware Support	Many cameras, including industrial ones, interfaces with its own custom hardware, optogenetics, external hardware integration and external software control (if supported).
Programming Model	Graph-based visual programming language.
Data Standardization	Various video formats allowed, export to Excel.
Comment	Has powerful animal tracking and integrated data analysis, similar to ANY-Maze.
Tool	**Bonsai RX**
Open Source	Yes, free
Synchronization Method	Manual / TTL Pulses
Hardware Support	Wide variety of modules for a lot of hardware available
Programming Model	Graph-based visual programming language, Python via IronPython (.NET flavor).
Data Standardization	Many file formats in a completely user-defined layout.
Comment	Extremely well integrated with OpenEphys hardware, established Open Source community.
Tool	**PyControl**
Open Source	Yes, free
Synchronization Method	Sync pulses via Rsync / camera frame trigger
Hardware Integration	Custom open source hardware for closed-loop interfacing & control

Programming Model	Needs external tools for hardware support, focuses on actuator control
Data Standardization	Custom format, Numpy data
Comment	Syntalos could make use of PyControl directly via its Python support, allowing to get the best out of both tools.

Tool	**AutoPiLot**
Open Source	Yes, free
Synchronization Method	Manual, TTL pulses
Hardware Integration	Purely Raspberry Pi based
Programming Model	Python
Data Standardization	Anything that Python can write with custom, user-written code.
Comment	Very young project based on a swarm of Raspberry Pi, very different concept compared to Syntalos.

Tool	**LabView**
Open Source	No, proprietary
Synchronization Method	Manual, sophisticated methods available with the right hardware / if implemented in G by the user (see https://www.ni.com/en/shop/labview/timing-and-synchronization-in-ni-labview.html)
Hardware Support	Variety of modules / device drivers available, National Instruments hardware is particularly well supported
Programming Model	Primarily graphical dataflow programming in G

Data Standardization	Many file formats in a completely user-defined layout.
Comment	Widely used if graphical programming is needed, but not open-source unlike Bonsai RX

Tool	**Simulink**
Open Source	No, proprietary
Synchronization Method	None, primarily used for running simulations.
Hardware Support	Support available as long as the hardware provides a MatLab interface
Programming Model	Graphical block diagram, Matlab code
Data Standardization	Many file formats in a completely user-defined layout.
Comment	Primarily used for simulations and to build hardware, and less for device control. Matlab skills are very helpful when using Simulink for data acquisition.
Tool	**OpenEphys GUI**
Open Source	Yes, free
Synchronization Method	Manual, TTL pulses
Hardware Integration	Only supports electrophysiology hardware and DSP processing elements, but includes a bridge to Bonsai to be used in conjunction with Bonsai.
Programming Model	Supports blocks that can be written in Python, or new blocks can be created in C++
Data Standardization	None.
Comment	Mostly focused on electrophysiological recordings, which it does very well. Can be combined with Bonsai to achieve similar features Syntalos provides [8].

Table S5: Devices tested with Syntalos

Electrophysiology	• Intan RHD2000 USB Interface Board
Miniscopes	• UCLA Miniscope v3<br>• UCLA Miniscope v4
The Imaging Source Cameras	• DFK 37BUX462<br>• DFK 37BUX290<br>• DMK37BUX287
Basler Cameras	• ace Classic acA1920-25um
Miscellaneous	• Arduino Uno Rev3 SMD<br>• Raspberry Pi Pico v1<br>• Raspberry Pi 4b

Table S6: Tetrode positions for electrophysiological aperture-detection experiment (Fig. 1)

**Tetrode**	**A/P with respect to Bregma [mm]**	**M/L [mm]**	**D/V with respect to dura [mm]**
BC (barrel cortex) 1	-1.0	3.25	-0.4
BC 2	-1.3	3.3	-0.3
BC 3	-1.6	2.7	-0.85
BC 4	-1.3	2.6	-0.85
BC 5	-1.0	2.7	-0.85

Table S7: Electrode positions for electrophysiological experiment shown in Fig. S7.

**Electrode**	**A/P with respect to Bregma [mm]**	**M/L [mm]**	**D/V with respect to dura [mm]**
OE (olfactory epithelium)	+ 3 mm from the nasal fissure	0.5	-1
mPFC (medial prefrontal cortex)	1.8	0.5	-2.2
dHPC (dorsal hippocampus)	-2	1.6	-1.4
vHPC (ventral hippocampus)	-3.1	3.3	-3.5

Supplementary References

1. Dragly, S.A. et al. Experimental directory structure (Exdir): An alternative to HDF5 without introducing a new file format. *Frontiers in Neuroinformatics* **12**, 1-13 (2018).
2. Lopes, G. et al. Bonsai: An event-based framework for processing and controlling data streams. *Frontiers in Neuroinformatics* **9**, 1-14 (2015).
3. Muller, E. et al. Python in neuroscience. *Frontiers in Neuroinformatics* **9** (2015).
4. Rübel, O. et al. The Neurodata Without Borders ecosystem for neurophysiological data science. *eLife* **11**, e78362 (2022).
5. Herz, A.V.M., Meier, R., Nawrot, M.P., Schiegel, W. & Zito, T. G-Node: An integrated tool-sharing platform to support cellular and systems neurophysiology in the age of global neuroinformatics. *Neural Networks* **21**, 1070-1075 (2008).
6. Gill, J.P., Garcia, S., Ting, L.H., Wu, M. & Chiel, H.J. *neurotic* : Neuroscience Tool for Interactive Characterization. *eneuro* **7**, ENEURO.0085-0020.2020 (2020).
7. Schindelin, J. et al. Fiji: an open-source platform for biological-image analysis. *Nature Methods* **9**, 676-682 (2012).
8. Buccino, A.P. et al. Open source modules for tracking animal behavior and closed-loop stimulation based on Open Ephys and Bonsai. *J Neural Eng* **15**, 055002 (2018).
9. Tort, A.B., Hammer, M., Zhang, J., Brankačk, J. & Draguhn, A. Temporal relations between cortical network oscillations and breathing frequency during REM sleep. *Journal of Neuroscience* **41**, 5229-5242 (2021).
